# Participation shifts explain degree distributions in a human communications network

**DOI:** 10.1371/journal.pone.0217240

**Published:** 2019-05-23

**Authors:** C. Ben Gibson, Norbou Buchler, Blaine Hoffman, Claire-Genevieve La Fleur

**Affiliations:** Army Research Laboratory, Aberdeen, Maryland, United States of America; Cinvestav-Merida, MEXICO

## Abstract

Human interpersonal communications drive political, technological, and economic systems, placing importance on network link prediction as a fundamental problem of the sciences. These systems are often described at the network-level by degree counts —the number of communication links associated with individuals in the network—that often follow approximate Pareto distributions, a divergence from Poisson-distributed counts associated with random chance. A defining challenge is to understand the inter-personal dynamics that give rise to such heavy-tailed degree distributions at the network-level; primarily, these distributions are explained by preferential attachment, which, under certain conditions, can create power law distributions; preferential attachment’s prediction of these distributions breaks down, however, in conditions with no network growth. Analysis of an organization’s email network suggests that these degree distributions may be caused by the existence of individual participation-shift dynamics that are necessary for coherent communication between humans. We find that the email network’s degree distribution is best explained by turn-taking and turn-continuing norms present in most social network communication. We thus describe a mechanism to explain a long-tailed degree distribution in conditions with no network growth.

## Introduction

Fundamental to the prediction of network phenomena is an explanation of heavy-tailed degree distributions—the enumeration of links among individuals in a network. Indeed, many observations of social networks and communication networks in particular is that the emergent degree distribution of emergent degree is “non-normal” and heavy-tailed [1]. In many systems, a few individuals dominate counts of network interactions and have very many links, whereas most individuals have just a few links. Researchers have observed such long-tailed, approximate Pareto-distributed degree distributions in a number of social networks [2–5], including human communication networks [6, 7]. The ubiquity of this observed approximate Pareto distribution has been of considerable interest to social scientists, as it deviates from Poisson-distributed counts that would normally be associated with random chance. Often, Pareto-distributed degree is explained by researchers via preferential attachment, which, under certain conditions, can create power law distributions. [8–12]; however, in conditions where the network has no growth in the number of nodes, preferential attachment instead converges to a complete graph [13]. Explanations of long-tailed degree in networks without growth thus still requires explanation.

A possible explanation for approximate power-law distributions in communication networks without growth could be conversational dynamics and norms present in many human interaction networks. A common process found in many social network communications is the existence of participation-shifts—a rules-based sequential shift in roles of speaker, recipient, and unaddressed recipient. Building upon previous models of conversation analysis, Gibson [14] asserts two propositions: first, that conversation is identifiable as an ordered phenomenon with rules governing who is to speak, and who is to be addressed; second, that conversation is comprised of actors with attributes (e.g. personality, hierarchical position, or other attributes) that affect the frequency and timing of their participation in the conversation. Without these two basic propositions, conversation would be chaotic, and successful communication impossible to achieve. *Participation shifts* are thus the sequential reshuffling of participation roles that depend upon the past history of the network. In the dyadic case, for example, a speaker, Person A, sends a communication event to Person B, who then responds. (The participation shifts framework was developed with a sender, recipient, and unaddressed recipient (audience) in mind as an implicit assumption. Unlike corporate meetings [14] or radio networks [15], email networks do not have an audience of the entire group at once, and side conversations—private communications between actors that are not seen by the group at large—are endemic. With this in mind, we test a pair of *recency* effects: RRecSnd (turn-taking with intervening events taking place) and RSndSnd (turn-continuing with intervening events taking place). Conceptualizing recency effects in addition to participation shifts allows intermediate events (emails) to occur between responses.). This participation shift is labeled AB-BA, in the typology developed by Gibson. See Table 1 for a list of all dyadic participation shifts. As a communication process continues, role shifts depend heavily upon the past history of the communication network series; e.g., the rate with which an individual is addressed in the past will affect their participation going forward. Without *p-shifts*, mutual intelligibility would be limited, and conversation incoherent [14, 16, 17]. These participation shifts are indeed a strong predictor of communication networks in a number of contexts; humans typically maintain turn-taking and turn-continuing dynamics in radio networks, corporate meetings, and classrooms [14, 15, 18].

**Table 1:** Dyadic participation-shifts (P-shifts).

Participation Shift	Explanation
PSAB-BA	Turn receiving: conversation event from *A* to *B* is immediately followed by conversation event *B* to *A* (special case of *RRecSnd*)
PSAB-BY	Turn receiving: conversation event from *A* to *B* is immediately followed by conversation event *B* to Person *Y*
PSAB-XA	Turn usurping: conversation event from *A* to *B* is immediately followed by conversation event *X* to *A*
PSAB-XB	Turn usurping: conversation event from *A* to *B* is immediately followed by conversation event *X* to *B*
PSAB-XY	Turn usurping: conversation event from *A* to *B* is immediately followed by conversation event *X* to *Y*
PSAB-AY	Turn continuing: conversation event from *A* to *B* is immediately followed by conversation event *X* to *Y* (special case of *RSndSnd*)
RRecSnd	Recency of conversation event from Person *A* to Person *B* affects Person *B*’s future rate of communicating with Person *A*
RSndSnd	Recency of conversation event from Person *A* to Person *B* affects Person *A*’s future rate of communicating with Person *B*

Our natural line of inquiry then is to investigate the link between the ubiquity of *p-shifts* in human communication dynamics and ubiquitous long-tailed degree distributions in these networks. For example, strong dyadic turn-taking norms could require most conversation participants to be silent while few actors respond to one another, causing most degree counts to be highly concentrated in just a few participants. Given the prevalence of this type of communication dynamic in human social networks, we expect email networks to have similar rules governing the participation framework over time. For email networks, p-shifts could explain why certain individuals send or receive more emails than others—through e.g., AB-BA (turn-taking dynamics), AB-AY (mass email events), or AB-BY (passing on information). With strong p-shift effects, those nodes that communicated early in the series could accumulate a higher degree by the continuation of past conversational dynamics into the future.

**Fig 1:**
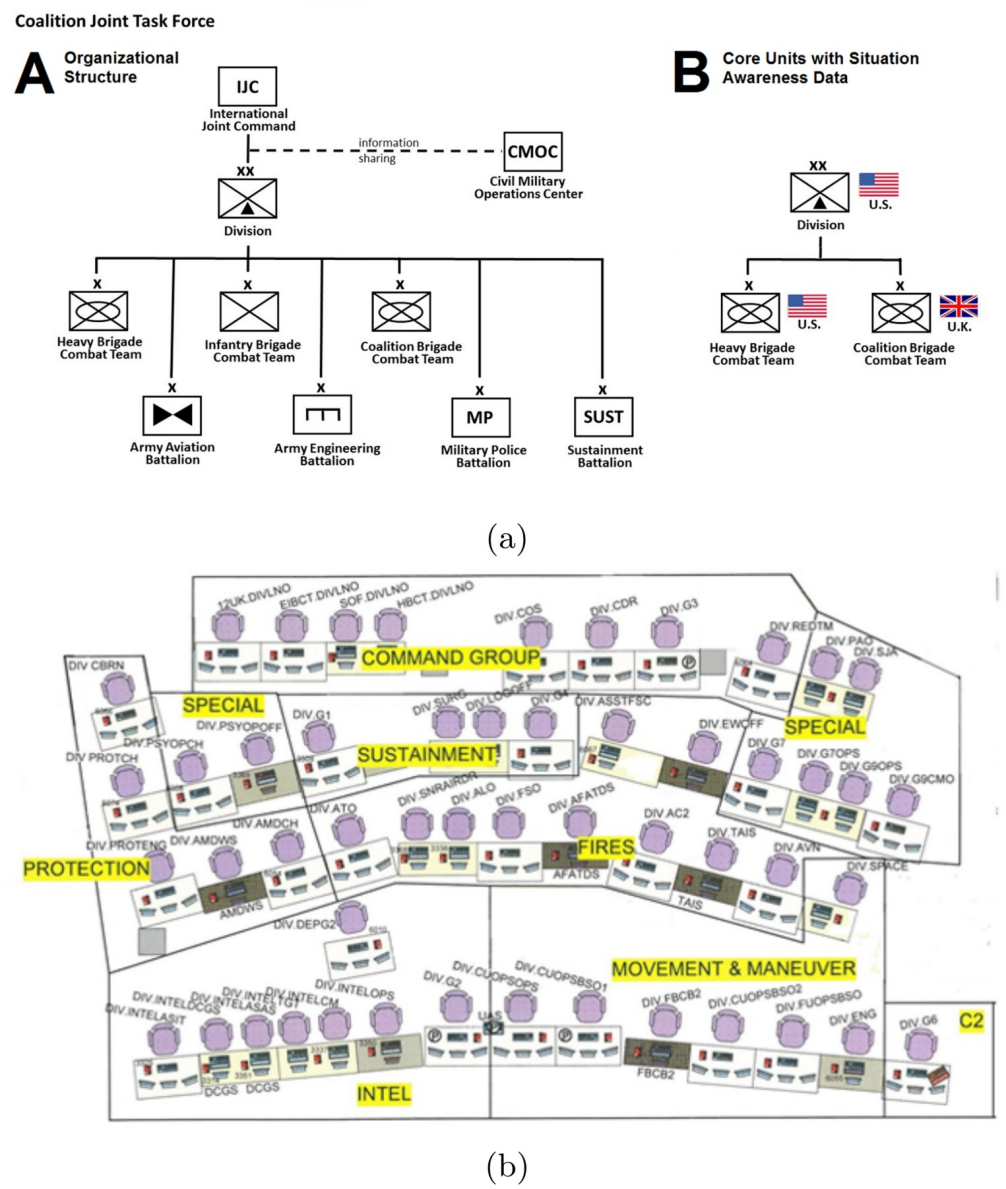
Coalition joint task force. (a) (left) Organizational structure of the Coalition Joint Task Force during the two-week military training exercise held at the Mission Command Battle Laboratory (Fort Leavenworth, Kansas). The network organization spans multiple echelons from Joint Command to Division to Brigade to support Battalions. (right) The core units exercised during the training event and subjected to our analysis- the Mission Command staff of a U.S. Division and two sub-ordinate Brigades, a U.S. Heavy Brigade Combat Team and a U.K. Coalition Brigade Combat Team. Individual situation awareness data was collected from the participating staff of these three core units. (b) Example seating chart for the Mission Command staff of the U.S. Division.

Shown in Fig 1, we analyze data collected from a hierarchical networked organization composed of active duty staff of the United States Army and United Kingdom as they participated in and conducted operations during a large-scale distributed military exercise at the Mission Command Battle Laboratory (Fort Leavenworth, Kansas) using a sophisticated simulation environment to drive scenario events. The key Mission Command staff consisted of three core units: a U.S. Division and two sub-ordinate Brigades; a U.S. Heavy Brigade Combat Team and a U.K. Coalition Brigade Combat Team.

## Materials and methods

Army Research Lab IRB approved the following study; verbal consent was granted by participants. The military organization addressed specific problems that occurred in simulation and during mission execution over a two week period as they conducted both military and civil-military operations. This dataset reflects the operations of a work-directed networked organization functioning as a *purposive social system* where staff members are readily known to one another by role and position and work collaboratively to accomplish one or more common objectives [19]. The responsibility for accomplishing the various tasks and sub-tasks were divided and assigned among the staff and included monitoring key events, analyzing information, adhering to work routines, developing work products, and coordinating an effective response, given resource limitations.

Our focus on a work-directed network organization differs from conventional network studies that are focused on self-perceived social relationships or structurally-defined social scenarios [20]. The data collection and analysis focused on all email activity (9,750 total messages) among all 94 participants involved in the two-week exercise. Only emails between participants were considered for study. Thus, our setting consists of a time-stamped communications network with self-directed emails as discrete ephemeral links between endpoints. This differs from conventional approaches that are based on social-structure as enduring self-reported or observed relationships. Participation shifts and preferential attachment of social actions by definition depend upon the history of social actions in the past. We model sequential participation shifts by incorporating the past history of the network, using relational events models (REM) [15]. We aim to test the efficacy of the preferential attachment (PA) versus participation shifts in predicting degree distribution.

Social network models typically treat network ties as having a persistent form. Friendship networks, sexual partnerships, organizational ties, etc., all are higher-level concepts that represent a meaningful label to the repeated interactions between actors. Typically, however, these social ties do have periodic interactions, or *social actions*. These social actions then combine to form a *gestalt* relationship, viewed by the participants and outsiders as having a meaningful form outside of the interactions themselves. Most network models, such as SAOMs, ERGMs, and DNR, aim to predict network structure by predicting the relationship itself, rather than the interactions that comprise these relationships [21, 22]. Here, we are interested specifically in the conversational dynamics between members of an organization, i.e., the social actions separate from their proscribed meanings.

Relational events modeling (REM) [15] uses the past history of social actions to predict the next actions in the sequence. Each action *a* has three elements, a sender *s*, a recipient *r*, and a timestamp *t*. In REM, social action dynamics are governed by the function:

$$\begin{array}{c} \lambda \left(s\left(a\right),r\left(a\right),X_{a},A_{t},\theta \right)=\exp\left[\lambda_{o}+\theta^{T}u\left(s\left(a\right),r\left(a\right),X_{a},A_{t}\right)\right] \end{array}$$ (1)

where

- λ_0_ is the baseline rate of social actions in the network.
- *X*_*a*_ are covariates
- *A*_*t*_ represents the past history of social actions.
- *u* is a vector of sufficient statistics.
- *θ* are the model coefficients associated with the corresponding *u*.

The function above is analogous to a “hazard analysis” (also called event sequence, or survival analysis) of instantaneous tie formation, where each event is governed by a hazard function with inputs from the past network history. It compares a baseline rate of social actions in the network with covariates associated with the past event sequence, i.e., elements associated with past tie formation. For a more complete treatment of relational events modeling, see [15]. We use Butts’ *relevent* package for our analysis [23].

The use of relational events modeling allows us to test the effects of both preferential attachment and participation shift dynamics as it relates to the observed degree distribution in the network. REM includes elements of the past communication sequence as a predictor of future communications: the extent to which, for example, high-indegree nodes participate more in future social activity. As an example using a p-shift, REM also allows us to measure the past participatory action sequence to spot email responses, either through the next email event in the sequence (AB-BA) or by a recency effect (RecRecSnd); it handles the AB-BY series by measuring whether the recipient of the action immediately in the past then becomes the sender in the next event. Through these parameters, we can predict which effects are responsible for the long-tailed degree distribution found in the network.

## Results

REM parameter results are listed in Table 2. The strongest are normalized indegree affecting future sending (NIDSnd) and receiving (NIDRec) rates. These are traditionally referred to as preferential attachment—effects that represent individuals being drawn into the conversation through repeated interactions. We note that though these are the strongest effects in the model, they do not recreate the exact degree distribution in simulations, as predicted by [13]. Second, normalized out-degree effects (NODSnd and NODRec) are strong for future sending rate, but not for future receiving rates. Though individuals may decide to send many messages into the network, it does not affect how many they receive in the future. Recency-receive effect (RRecSnd) is a dyadic-level effect that puts a rank-ordered response priority on recency of messages sent to person *i* from others in the network. For example, the last person to send a message to person *i* is scored a 1, the second-to-last person to send person *i* a message is scored a 1/2, and so on. Recency-send effect (RSndSnd) is a rank-ordered send priority from *i* to *j* when *i* has already sent emails to *j* in the past. Both of these effects have strong, positive coefficients in prediction of the next event in the series. Individual-level payrank and situational awareness also affected send rates positively; payrank was associated with future receive rates, but situational awareness was not. The greater the difference in payrank between actors, the more priority for response the lower-payrank actor gave to higher-payrank actors’ emails. Actors with lower situational awareness (SA) sent emails more often to those of higher SA than to those with the same or lower SA. As discussed above, most of the tested dyadic p-shifts were found to be significant and positive, the most powerful one being the AB-AY shift (the AB-AY shift also includes group emails). Residual deviance was 95712.11 on a null deviance was 173523.1 (AIC 95748.11).

**Table 2:** Relational events model of an email network among a command-and-control military exercise. Signif. codes: *p* < 0***; *p* < 0.001**; *p* < 0.01*.

Parameter	Estimate	Std Err	Z value	Pr	sig.
NIDSnd	12.93	1.24	10.44	0	***
NIDRec	25.00	0.92	27.1	0	***
NODSnd	13.56	0.40	34.02	0	***
NODRec	-0.61	0.42	-1.46	0.14	
RRecSnd	1.19	0.04	30.09	0	***
RSndSnd	3.12	0.04	70.77	0	***
Payrank: Send	0.42	0.09	4.48	0	***
SA: Send	0.35	0.14	2.39	0.02	*
Payrank: Receive	0.47	0.06	7.79	0	***
SA: Receive	-0.09	0.10	-0.9	0.37	
Rank Difference * Recency	0.01	0.00	15.2	0	***
Payrank Homophily	0.04	0.04	0.99	0.32	
SA Difference	1.07	0.03	38.57	0	***
PSAB-BA	2.05	0.12	17.08	0	***
PSAB-BY	0.70	0.11	6.4	0	***
PSAB-XA	0.06	0.12	0.5	0.62	
PSAB-XB	0.29	0.12	2.4	0.02	*
PSAB-AY	5.25	0.03	184.96	0	***
Null deviance	173531.1				
Residual deviance	95712.11				
AIC: 95748.11					

Though many parameters were strongly predictive of the relational events sequence in this network, only a few approached the degree distribution found in the data: PSAB-BY, PSAB-BA, and recency effects (RRecSnd and RSndSnd). (The participation shifts AB-BY and AB-BA are special cases of the recency effects listed here). Surprisingly, our in- and outdegree distributions were less heavy-tailed than preferential attachment would predict (i.e., what would be expected given on NIDSnd and NIDRec in the model). This is a somewhat different result as predicted in [13], which predicted a complete network with no network growth; we suspect that given a longer time series, their predictions may stand. As described above, our results suggest that participation shifts are responsible for the degree distributions in our network.

We find substantial effects for both preferential attachment and participation shifts in predicting social actions in this communication network. Past normalized indegree (received emails) greatly affects future participation in the network (outdegree), as well as future indegree. As participants are drawn into conversation in the network, the more they participate. This result highlights the kinetics of human networked communications. Among effects for participation shifts, turn-continuing (so-called AB-AY p-shifts) was the strongest predictor of future email behavior, while turn-receiving (AB-BY and AB-BA) were also strong indicators of future social actions. Similarly, recency effects were also strong, suggesting that email responses were prioritized by how recently the messages were received. As expected, normative conversational behavior is prevalent in this network series, confirming findings in other contexts. [14, 15, 18]

Individual-level attributes also had significant effects on email behavior. Those of higher payrank—a cross-comparable measure of social capital based on published monthly salary taking into account both military rank and years of service—had higher hazard for both sending and receiving emails. We also measured the situational awareness of each individual to ongoing events as a daily pop-quiz [24]; those with higher measures of situational awareness during the exercise had higher hazard of sending emails but not receiving them. A small interaction effect for recency by payrank suggests that actors were slightly more likely to respond faster to emails received from higher-ranked actors than others.

We are primarily interested in how preferential attachment and participation shifts relatively affect the in- and out-degree distributions observed in our network. Using relational events modeling, we build minimal models for each theory, each with its own covariate set. Our intention is to discover the simplest models needed to reproduce the observed degree distribution. We first included two measures of preferential attachment: normalized indegree affecting future rate of sending; and normalized indegree affecting future rate of receiving. Both use the past indegree in predicting future activity in the network, which reflects a preferential attachment process. Second, we consider only effects for participation shifts, one by one, in separate relational events models. Parameters are estimated in each model fit, which are then used to predict the email network. We compared predicted degree distributions with the observed distributions using the Kolmogorov-Smirnov test [25].

**Fig 2:**
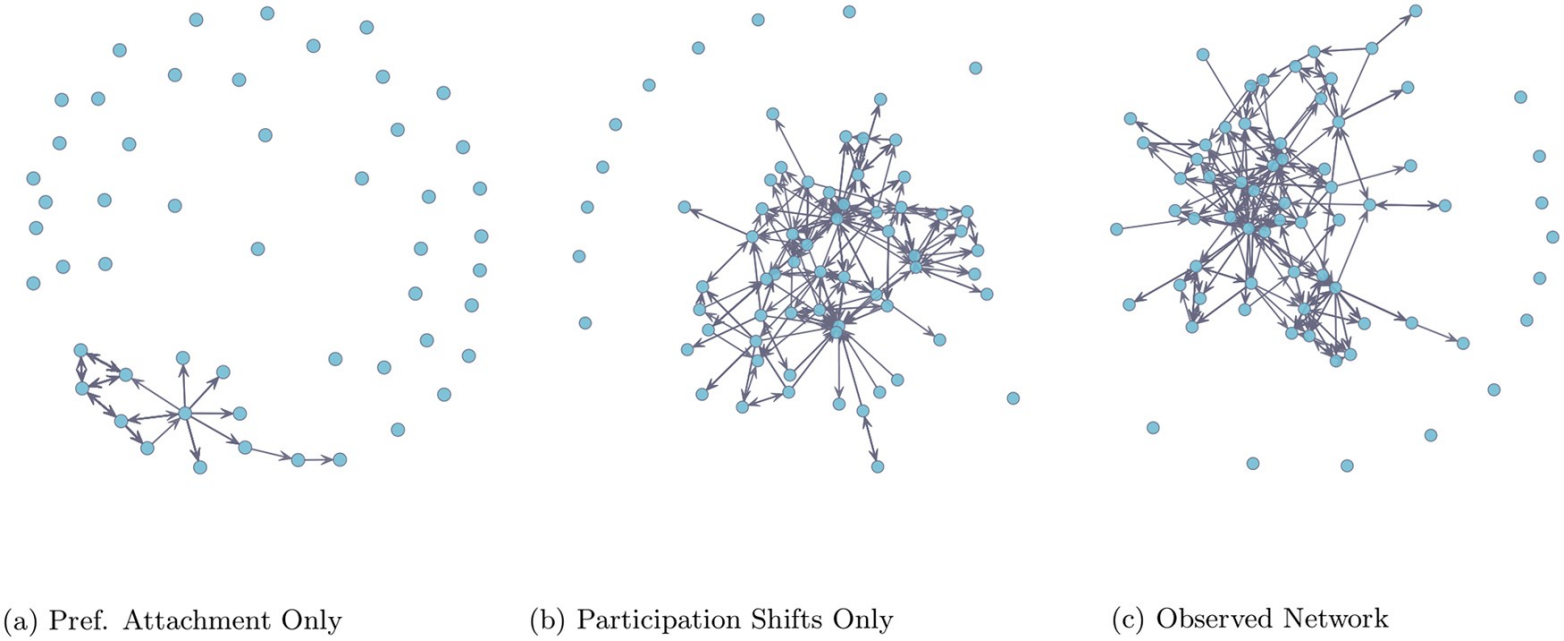
Predicted network comparisons. Preferential attachment and participation shifts as explanations of network structure in observed data. Figs (a)-(b) are simulated email networks using fitted parameters from a model including only preferential attachment and participation shifts, respectively. The network structure in simulated network (b) tends to resemble the observed network (c), though (a) is the typical explanation of long-tailed degree structure. 300 email events were simulated among 94 nodes.

We find that two classes of participation shifts predict the long-tailed in- and outdegree distributions in our network. The primary driver of the indegree distribution was dyadic turn-taking dynamics (AB-BA, see Fig 2a). Turn-taking dynamics are a key aspect of coherent communications in human social networks with multiple participants [14, 15, 26]. The best model for prediction of the outdegree distribution was the turn-continuing shift (AB-AY). In an email network, this includes instances where an individual sends emails in bursts, a common feature of communication networks [27]. Researchers [28] maintain that human communication patterns are “bursty” as the inter-event arrival times between messages tends to follow a power-law distribution with short intervals between many messages but yawning gaps between others. In a model combining these two effects, we find that the predicted and observed degree distributions are not significantly different, suggesting that these two parameters alone are sufficient to produce the observed distributions found in our network.

**Fig 3:**
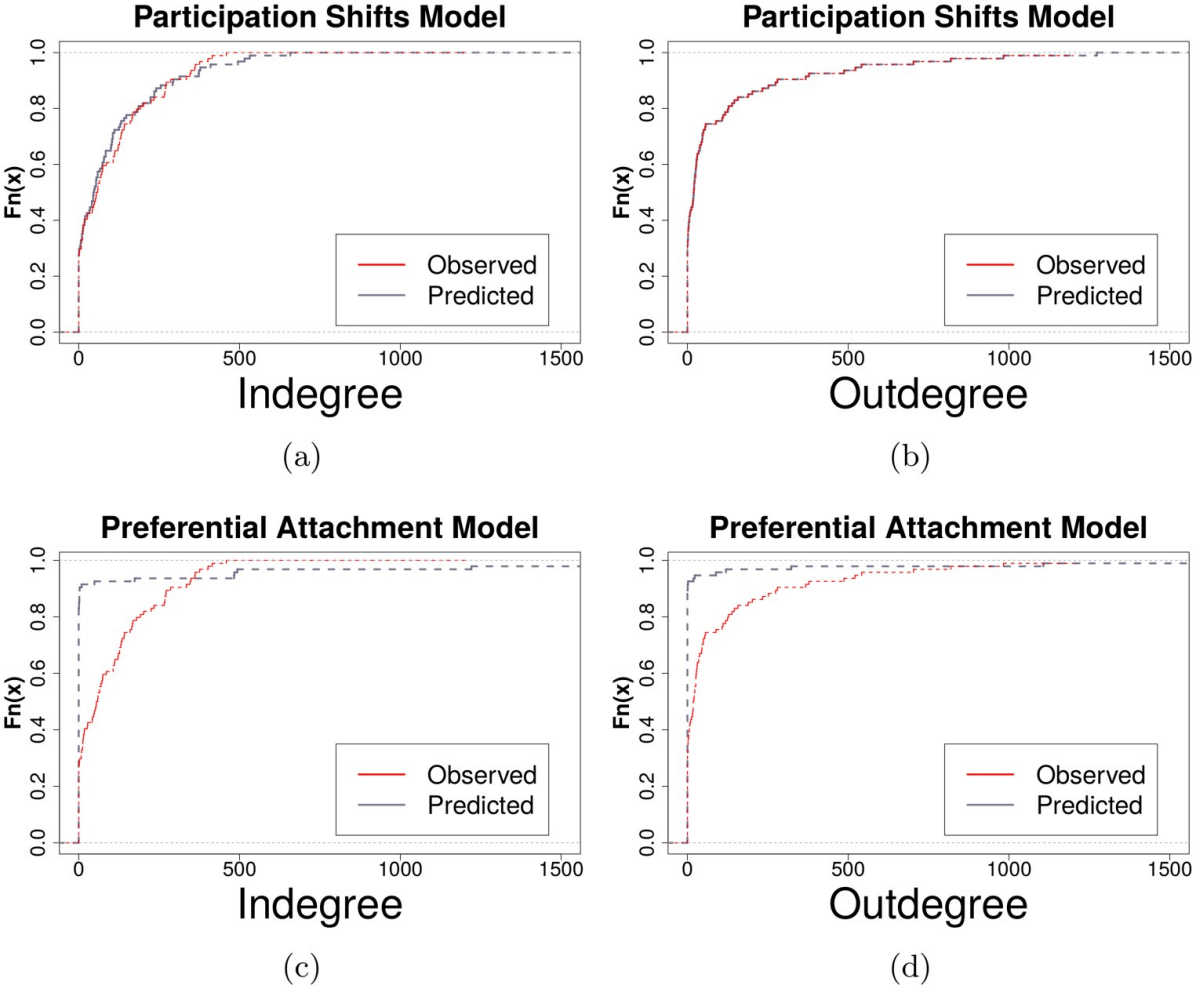
(a) Predicted indegree distribution by participation shift type. Each prediction is drawn from a separate relational events model including only the participation shift shown. PSAB-BA best predicts the indegree distribution, but is not fully adequate to do so. (b) Predicted outdegree distribution by participation shift type. PSAB-AY participation shift perfectly reproduces the outdegree distribution without any other parameter.

**Fig 4:**
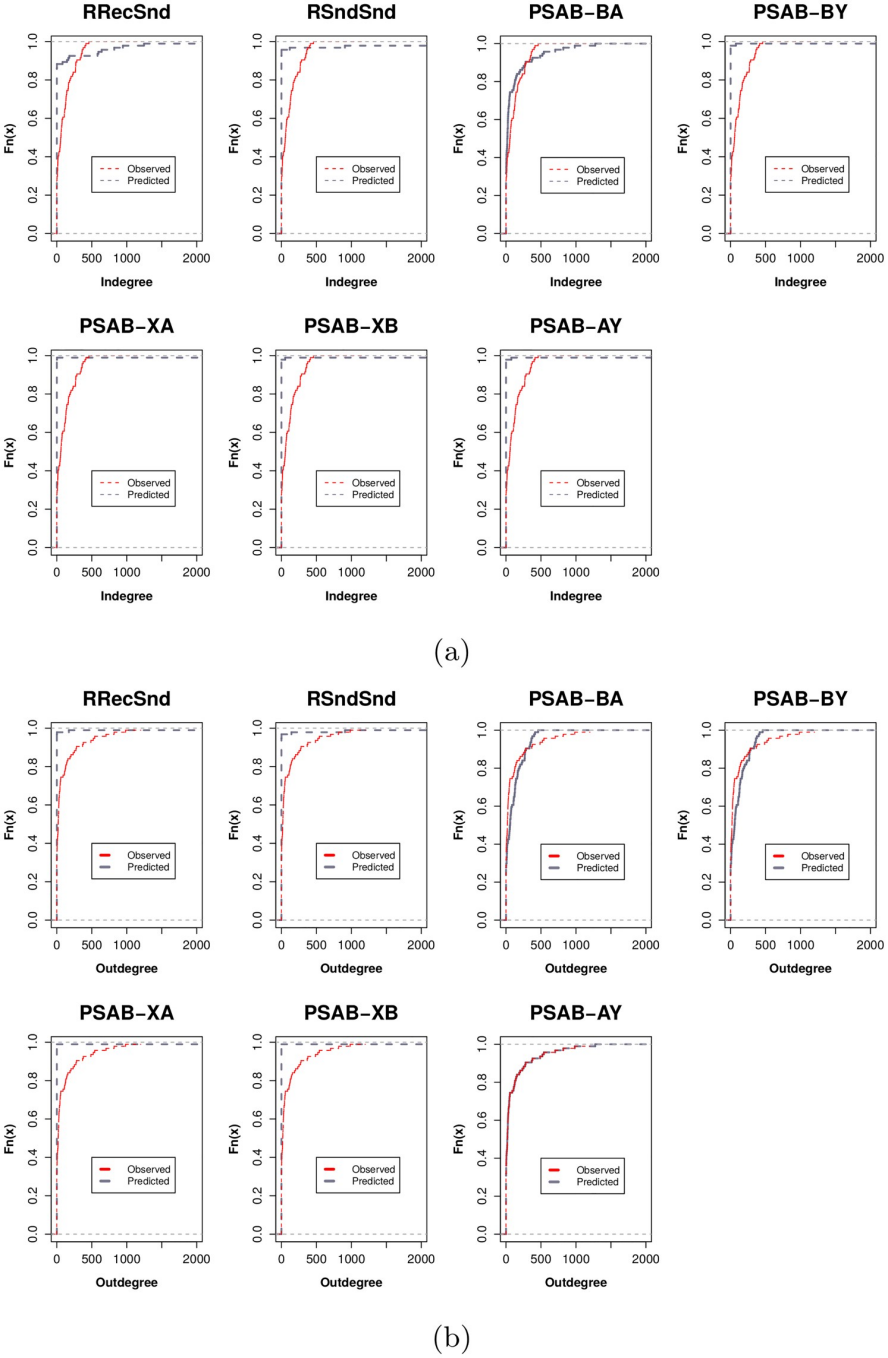
Relational events modeling of email comms. Parameters from a relational events model predicting sequential, dyadic communicative actions between actors in the network. The strongest effects (NIDSnd and NIDRec) represent preferential attachment, as those with greater indegree participate more in the future. The next strongest class of predictors are “burstiness” parameters (NODSnd and PSAB-AY). The effect size for PSAB-BA participation shift (turn-taking dynamics) is relatively smaller, but strong.

We also test whether models including preferential attachment reproduce the network’s distributions (see Fig 3). According to [13], the network series should converge on a complete network given no growth and preferential attachment. There are strong effects for preferential attachment (see Fig 4), as those with high levels of normalized indegree greatly affect future participation rates. Though the effects are strong, predictions from models only including preferential attachment produce degree distributions significantly different from the ones found in the observed network (KS test *p* < .001). The prediction that preferential attachment will converge on a complete network did not occur, it may have converged on a long enough time series. Here, the effects of preferential attachment were such only a very few nodes had any degree at all. While this does contradict previous work, future studies should determine whether these results will hold in other communication networks.

## Discussion

**Fig 5:**
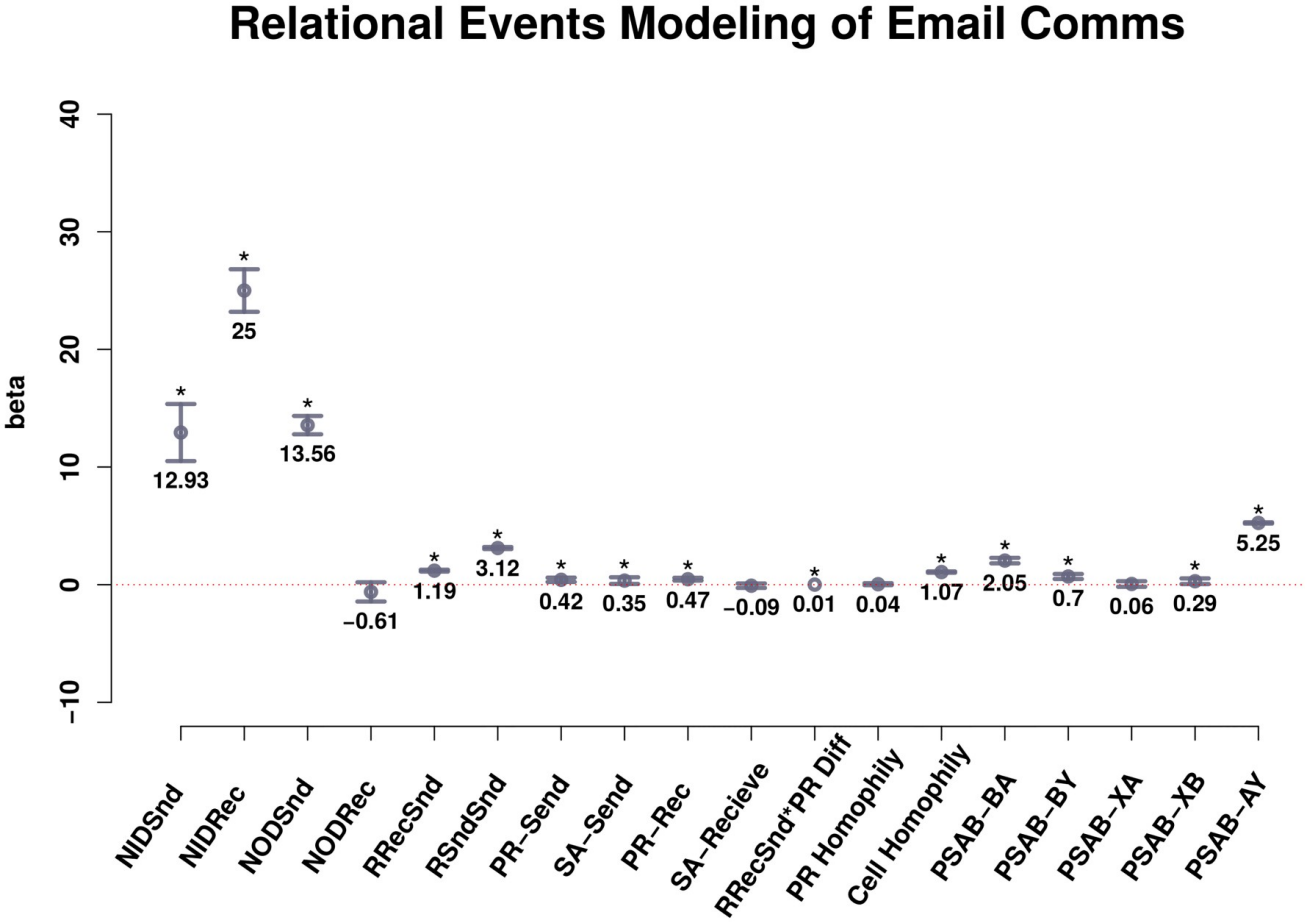
(a)-(b) CDF of observed versus predicted degree distributions (via relational events model) given only selected participation shifts (AB-BA, AB-BY, RecRecSnd) in the model. Predicted degree distributions match that of observed (KS test *p* > .05). (c)-(d) CDF of observed versus predicted degree distributions given only preferential attachment. Predicted degree distributions (via relational events model) did not match observed (KS test *p* < .001).

Our models show many possible candidates for prediction of the degree distribution, as we found many significant ecological and individual-level attributes predicting communication dynamics (see Fig 4). However, out of all explanations we tested, dyadic turn-taking dynamics and turn-continuing dynamics best explain the long-tailed degree distributions found in an observed communications network. These two concepts—turn-taking and turn-continuing—are essential elements of coherent communications between humans [14, 16, 17]. In some cases, turn-continuing p-shifts are referred to as “burstiness”, and have been used as an explanation of other long-tailed distributions such as response waiting times in communications networks [27]. The prevalence of these participation-shifts in social networks, combined with the prevalence of their long-tailed degree distributions, suggests a possible implicit link that should be investigated further using other communication network settings. Actor-level normalized indegree affecting future participation rates had strong effects in our model, but did not reproduce observed in- and outdegree distributions (see Fig 5(c) and 5(d)), as predicted in [13].

Other work has focused on preferential attachment plus growth that results in approximate power laws for tie distributions [10]; a key distinction between the network described here is that during our natural experiment, the set of nodes remains fixed, and no growth was observed. Additionally, this network is relatively small compared to other studies, which makes it more suitable for the computational load required by network analytic methods like REM. This paper thus provides a mechanism for approximate Pareto-distributed degree where growth is not required, and the network is small. Future work should investigate if these results hold in a other communication networks, especially those with a growing number of actors, and in larger networks.

A final insight from this analysis is the success of an ecological factor (norms necessary for inter-human communication) in predicting an ecological outcome (the shape of the degree distribution across actors in the network). In our network, no individual-level factors were necessary in predicting the degree distribution; if the p-shift parameter sizes were held constant, individuals are completely interchangeable with regards to their effects on the degree distribution. Indeed, some aspects of human activity emerge from groups of individuals, and the prediction of this activity should then include aspects independent of the composition of individuals within [29].

## Conclusion

Long-tailed degree distributions are found among many social phenomena. Preferential attachment is the most common explanation, but have limitation in networks with a static number of nodes. We find that participation shifts—turn-taking and turn-continuing participation norms found in nearly all measured human communication networks—predicts degree distributions that match those of the observed network with an unchanging number of nodes. The prevalence of participation shifts in communication networks provides a viable explanation of long-tailed degree in many observed social networks, and should be considered in further investigations of similar settings.

## Supporting information

S1 File This is a file containing ego (i.e., participant) information. The files include variables needed to replicate the analysis. These can be matched with S2 File for analysis of edge dynamics.
(CSV)

S2 File This is a file containing edge (i.e., email) information. These can be matched with S1 File for analysis of edge dynamics.
(CSV)
